# The sex effect on balance control while standing on vestibular-demanding tasks with/without vestibular simulations: implication for sensorimotor training for future space missions

**DOI:** 10.3389/fphys.2023.1298672

**Published:** 2024-01-08

**Authors:** Li Zhang, Chao Qin, Jung Hung Chien

**Affiliations:** ^1^ Department of Rehabilitation Medicine, The First Affiliated Hospital of Guangxi Medical University, Nanning, Guangxi, China; ^2^ Department of Neurology, The First Affiliated Hospital of Guangxi Medical University, Nanning, Guangxi, China; ^3^ Independent Researcher, Omaha, NE, United States

**Keywords:** vestibular stimulation, sensory organization test, balance control, standing, mastoid vibration

## Abstract

**Background:** Anatomical differences between sexes in the vestibular system have been reported. It has also been demonstrated that there is a sex difference in balance control while standing on vestibular-demanding tasks. In 2024, NASA expects to send the first female to the Moon. Therefore, to extend the current knowledge, this study attempted to examine whether different sexes respond differently to vestibular-disrupted and vestibular-demanding environments.

**Method:** A total of fifteen males and fifteen females participated in this study. The vestibular function was quantified through different SOT conditions (SOT1: baseline; SOT5: vestibular demanding by standing with blindfolded and sway reference surface). The vestibular stimulation (VS) was applied either unilaterally or bilaterally to vestibular system to induce the sensory-conflicted and challenging tasks. Thus, a total of 6 conditions (2 SOT conditions X 3 VSs: no-VS, unilateral VS, and bilateral VS) were randomly given to these participants. Three approaches can be quantified the balance control: 1) the performance ratio (PR) of center of gravity trajectories (CoG), 2) the sample entropy measure (SampEn) of CoG, and 3) the total traveling distance of CoG. A mixed three-way repeated ANOVA measure was used to determine the interaction among the sex effect, the effect of SOT, and the effect of VS on balance control.

**Results:** A significant sex effect on balance control was found in the PR of CoG in the anterior-posterior (AP) direction (*p* = 0.026) and in the SampEn of CoG in both AP and medial-lateral (ML) directions (*p* = 0.025, *p* < 0.001, respectively). Also, a significant interaction among the sex effect, the effect of SOT, and the effect of VS on balance control was observed in PR of CoG in the ML direction (*p* < 0.001), SampEn of CoG in the AP and ML directions (*p* = 0.002, *p* < 0.001, respectively), and a traveling distance in AP direction (*p* = 0.041).

**Conclusion:** The findings in the present study clearly revealed the necessity to take sex effect into consideration while standing in vestibular-perturbed or/and vestibular demanding tasks. Also, the results in the present study could be a fundamental reference for future sensorimotor training.

## 1 Introduction

On Earth, humans are capable of detecting gravity, orienting themselves to their surroundings, and performing sensory motor activities, such as walking and standing in the dark or on slippery, uneven, or foamy surfaces, without any hesitation. A vestibular system plays an important role in detecting head rotation, acceleration, and self-motion transitions in relation to gravity in the above-mentioned circumstances (Messina et al., 2021). Also, when the head moves, the vestibular ocular reflex maintains the stability of the eyes in inertial space, so that the retinal image of the fixed visual surround appears to be stable, regardless of the motion of the head (Martines et al., 2021). It should be noted, however, that standing or walking in microgravity or at varying levels of gravity may result in different vestibular responses to maintain balance control. A prime example is Dr. Harrison Schmitt, an astronaut on the Apollo 17 mission, who fell several times and encountered difficulties in getting up from the Moon’s surface due to the fluctuating levels of gravity while picking samples (https://www.youtube.com/watch?v=Ke65jU_yYso, assessed on 5 November 2023). Moreover, Jain et al. (2010) and Wood et al. (2015) examine vestibular-related balance control in 11 crew members prior to and after spaceflight (11–13 days short-duration shuttle spaceflights) and suggest that alternate gravity temporarily impairs the vestibular system, which causes misinterpretations of the central nervous system and causes imbalances when astronauts stand or walk. The question may arise as to whether there is a sex difference in vestibular function when standing in a vestibularly disrupted environment.

Males and females have physiological and anatomical differences when it comes to the vestibular system (Bowman et al., 2000; Corazzi et al., 2020; Lien and Yang, 2021; El Khiati et al., 2022). In particular, it has been shown that female cochlea is shorter than male cochlea, resulting in a stiffer basilar membrane (Corazzi et al., 2020). Consequently, this anatomical sex difference in the basilar membrane may further influence the perception of vestibular signals (Bowman et al., 2000). It has been demonstrated that females exhibit higher amplitudes of ocular vestibular-evoked myogenic potentials and superior horizontal semicircular canal function than males, which results in a higher sensitivity to vestibular signal perception in females than males under air-conducted sound, bone-conducted vibration, and galvanic vestibular stimulation (Sung et al., 2011; Battersby, 2019; Schubert et al., 2022). In females, this increased sensitivity to vestibular-related perception may lead to the possibility of an imbalance when performing vestibular-demanding tasks, such as standing on a foam surface and closing their eyes. Vereeck et al. (2008) and Wolfson et al. (1994) support this abovementioned hypothesis by showing that the sex effect (302 females vs. 250 males) has a significant effect on the vestibular system in controlling balance while standing in different vestibular-demanding environments. In particular, numbers of females adopt a stepping strategy (step over the platform because of losing balance). In general, females demonstrated worse balance control than males while standing in vestibular-demanding tasks, such as standing with eye-closed and a single leg on the foam surface, standing with eye-closed and both legs on the foam surface, or standing with eye-closed and both legs on sway-reference surface (Sensory Organization Test condition 5, SOT5). NASA plans to send its first female astronaut to the Moon in 2024 as part of its Artemis program and plans to send more female astronauts to space missions in the years to come. According to the studies, it seems that females have a tendency to be fall while standing in a vestibular-demanding environment. It is essential to gain a better understanding of how the vestibular-related balance control differs in men and women when standing in a disrupted vestibular environment in order to develop sensorimotor training programs that will prevent falls among female astronauts in the future.

As part of clinical assessment (Black et al., 1989; Goebel and Paige, 1989; Hytönen et al., 1989; Mulavara et al., 2013), the SOT has frequently been employed to assess the vestibular function in patients with various vestibular disorders by simultaneously disturbing both the visual system (closed eyes) and the somatosensory system (sway-reference surface). Despite being constructed approximately 30 years ago, this SOT remains in use in the present time to assess vestibular function in astronauts immediately following spaceflight (11–13 days short-duration shuttle spaceflights, Ozdemir et al., 2018) and to assess whether 48 astronauts recovered balance control after prolonged spaceflight (approximately 6 months, Tays et al., 2021; Shishkin et al., 2023). According to the findings, staying in microgravity for a long period of time requires at least 30 days in order to fully restore balance control due to the adaptation and re-adaptation of the vestibular system between different level of gravity (Tays et al., 2021; Shishkin et al., 2023). For SOT, the equilibrium scores are used to quantify the vestibular function by measuring the movement of the center of gravity primarily forward and backward (Black et al., 1989; Goebel and Paige, 1989; Hytönen et al., 1989; Mulavara., 2013; Vanicek et al., 2013). Specifically, the limit of stability is approximately seven degrees (posteriorly) and five degrees (anteriorly, Vanicek et al., 2013). Participants who step off from the platform will receive an equilibrium score of 0 (failure equilibrium score, Vanicek et al., 2013). In this regard, however, it raises three concerns about this measure of equilibrium score: 1) the ceiling effect, 2) the sensitivity, and 3) the neglect of balance control in the ML direction. Also, it is important to note that even in patients with known vestibular lesions, the measure of equilibrium score in SOT is only about 50% sensitive to vestibular loss (Mishra et al., 2009). It is therefore important to use the equilibrium score measure cautiously in the evaluation of vestibular function since it may result in an incorrect interpretation of the sex effect on vestibular-related balance control. It may explain why a study have found no statistical differences between sexes in vestibular-related balance control using the measure of equilibrium score in SOT (Faraldo-Garca et al., 2011). Interestingly, this study still suggest that the sex effect should be taken into account when determining vestibular function because women use ankle strategies more than men when performing a vestibular-demanding task (Faraldo-Garca et al., 2011). Therefore, one of aims in present study was to assess balance control using other three practical measurements rather than the equilibrium score to determine the sex effect on vestibular system as follows: performance ratio of movement in the center of gravity (CoG), total traveling distance of CoG, and the complexity of movement of CoG using entropy measure.

The performance ratio (PR) is used to identify the degree of balance control (center of gravity, CoG) sway in the anterior-posterior (AP) and medial-lateral (ML) directions and is calculated by the numerical integral of the rectified CoG sway signal scaled to be a fraction of the maximum sway amplitude while standing on normal or perturbed environment (Nashner et al., 1982). This PR has been used to differentiate the patients with different types of vestibular deficits (Black et al., 1989; Nashner and Peters, 1990). In short, a greater PR reflects a greater sum of instantaneous sway, indicating a greater reliance on the vestibular system to maintain balance while performing a vestibular-demanding task. The total traveling distance of the CoG can simply be defined as the distance the CoG travels in a given period of time. A longer traveling distance commonly be interpreted as a worse balance control. For over a decade, the concept of entropy has been widely used to describe the complexity of physiological signals through time series analysis (Richman and Moorma, 2000; Richman et al., 2004; Donker et al., 2007; Hansen et al., 2017; Montesinos et al., 2018; Blazkiewicz et al., 2021; Fischer et al., 2023). Compared to conventional calculations in sway changes in means and standard deviations, analyzing the sway data in time series can provide another aspect of balance controls in uses in degree of freedom and understand the underlying causes of trends or systemic patterns over time. A sample entropy (SampEn) method has the following advantages: 1) SampEn has a better data length independence, 2) SampEn has better anti-noise capacity, and 3) SampEn is suitable for short datasets (Richaman and Mooram, 2000; Montesinos et al., 2018). In light of the differences in SampEn values, it is likely that executing different types of movements in time series requires a different degree of freedom. A more irregular movement (greater SampEn value) is commonly observed for adapting to the complex (sensory-conflicted) environment (Jia et al., 2017; Chen et al., 2021). In particular, patients with various vestibular dysfunction, no matter whether they stand on a solid or compliant surface, have a greater SampEn value than healthy controls (Lubetzky et al., 2018). Also, in these patients with vestibular dysfunction, SampEn values are even greater when standing on compliant surfaces than on solid surfaces (Lubetzky et al., 2018), requiring higher degree of freedoms in movements to control balance.

A large, well-developed facility such as NASA, for example, has the capability of measuring the changes in vestibular-related balance control between sexes in response to gravity changes; however, these costs cannot be justified in a typical biomechanical laboratory. There is, however, a feasible and cost-effective method of determining sex differences in vestibular-related balance control under vestibular-perturbed and vestibular-demanding environment by using vestibular stimulation (Kavounoudias et al., 1999; Lin et al., 2022). Specifically, applying VS increases the CoG sway area in both young (Lin et al., 2022) and older adults (Lin et al., 2022); furthermore, applying bilateral VS increased even more CoG sway area than applying unilateral VS in older adults (Lin et al., 2022), indicating that different types of VS induced different balance controls. The use of such a VS paradigm would mimic an unpredictable and vestibular-perturbed environment, which necessitates greater reliance on the vestibular system for the maintenance of balance. Thus, one of the aims of this study was to apply this paradigm to determine the sex differences in vestibular system for maintaining the vestibular-related balance control.

With the use of VS and SOT, this study was supported by NASA and attempted to meet NASA’s current focus on understanding the sex effect on vestibular-related balance controls while standing in normal and vestibular-demanding environments. This study expected to observe that 1) a sex effect was found on balance control, regardless of whether VS was administered or what SOT conditions participants were in, with females achieving worse balance control but experiencing higher degrees of freedom of control; 2) whether or not VS was given, there was a interaction between sex effect and SOT condition effect on balance control, 3) no matter what SOT conditions participants stood in, the balance control of females and males responded differently to VS, and 4) while standing in vestibular-demanding and bilaterally vestibular-perturbed condition (the most challenging task), female might demonstrate worse balance control and greater levels of degree of freedom in balance control than males compared to other conditions.

## 2 Materials and methods

### 2.1 Participants

In total, thirty healthy participants attended in this study (15 males and 15 females, age: 34.93 ± 17.36 years old, height: 170 ± 7.19 cm, weight: 68.17 ± 12.13 kg). To recruit these healthy adults, a variety of advertising methods were employed, including flyers posted on university campuses and in local community centers, as well as an online bulletin posted on the university’s website. All healthy adults were required to meet the following inclusion criteria: 1) all participants must be free of musculoskeletal deficits and have no history of extremity injuries, 2) participants must not have any joint surgeries that would affect their gait pattern, 3) participants must pass the dizzy handicap inventory (score = 0), indicating that potential vestibular dysfunction may not exist, and 4) participants never experienced any type of vestibular stimulation. The exclusion criteria were that 1) these healthy adults had any type of vestibular diseases or vestibular surgeries, 2) these healthy adults had any type of neurological disorders, and 3) participants scored below 23 in the Mini Mental State Examination, indicating the potential cognitive impairments (Foreman et al., 1996). This study was approved by the Institutional Review Board at the University of Nebraska Medical Center (IRB Protocol # 379-17-EP). The data collection only began while participants voluntarily signed the inform consent.

### 2.2 Experimental setup

A Balance Master System 8.4 (NeuroCom International Clackamas, OR, United States) was applied to identify the vestibular function through the sensory organization test. This system included a moveable visual surround and a support surface that could rotate around the ML axis led participants to lean forward (the maximum range was approximately 7°) or backward (the maximum range was approximately 5°) in the AP direction (Nashner and Peter, 1990). Two force plates (22.9 cm × 45.7 cm) were connected by a pin joint and used to record the displacement of center of gravity (CoG) at the sampling frequency of 100 Hz. The sensory organization test (SOT) contained a total of 6 conditions to identify the functions of visual, somatosensory, and vestibular systems (Black et al., 1989). In this study, only SOT-1 (baseline, stationary-platform, full visual support), and SOT-5 (sway-reference platform, eye-closed) were used to identify the effect of different vestibular stimulations on balance control during standing in normal and the vestibular-demanding tasks. Specifically, this SOT-5 has been widely used to probe the vestibular contributions to balance control in healthy controls (Hamid et al., 1991; Ford-Smith et al., 1995), in older adults and fallers (Horak et al., 1989), in patients with vestibular dysfunction (Di Fabio, 1995), and in astronauts (Tays et al., 2021; Shishkin et al., 2023).

**FIGURE 1:**
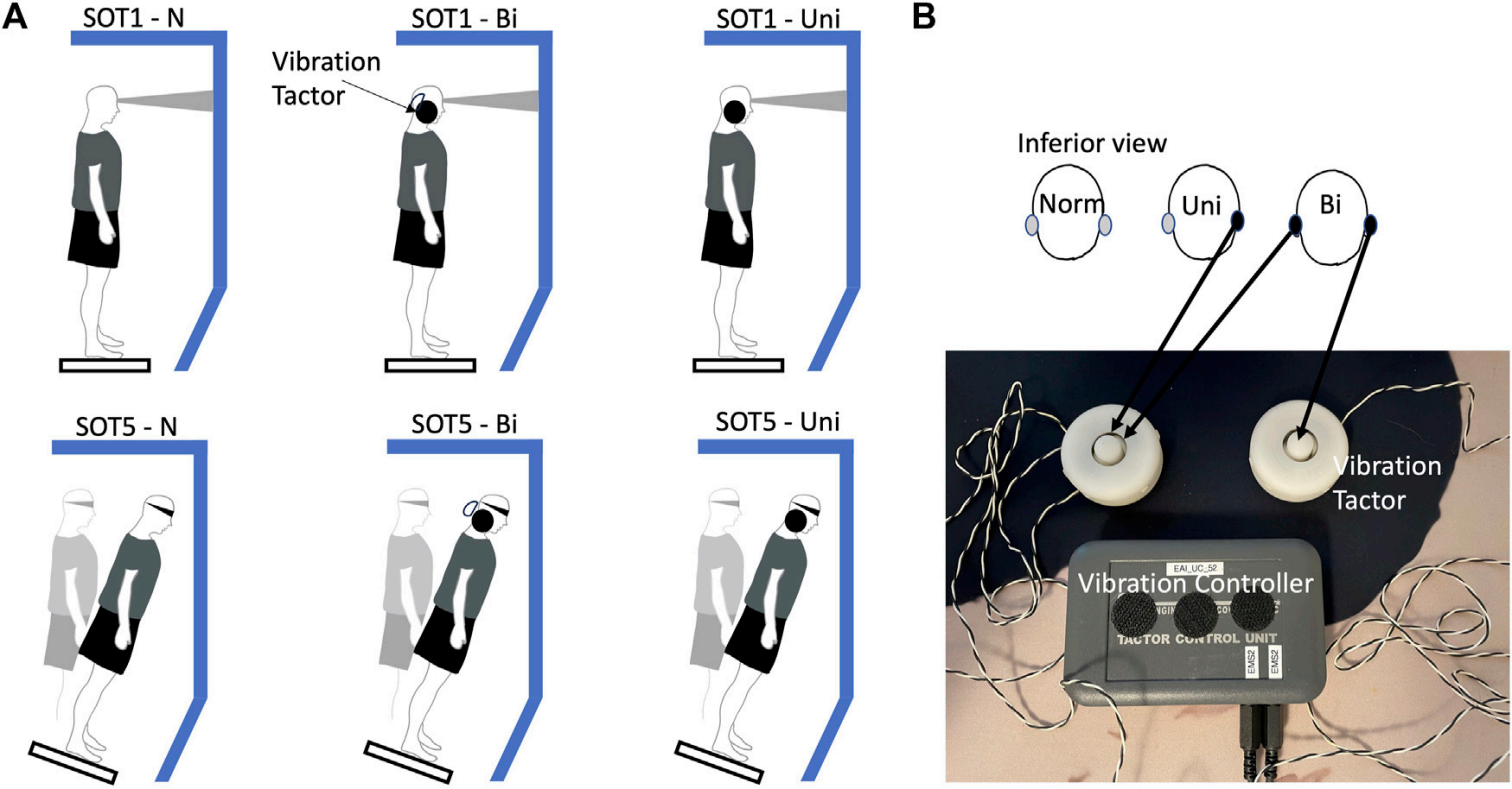
The experimental paradigm **(A)** the sensory organization test **(B)** the vestibular stimulation equipment.

In this study, mechanical vibrotactile stimulation (VS) was generated by placing two electromechanical vibrotactile transducers (EMS2 tactors; Engineering Acoustics, FL, United States) to the mastoid processes bilaterally. Participants can readily perceive the vibrations that occur through the use of these tactors, since they are designed to be mounted within a seat or cushion. With a rise time of 25 milliseconds, the EMS2 tactor produces large displacements even when applied against the mechanical impedance of the body. A maximum peak-to-peak displacement of 2 mm was recorded when the device was loaded. Tactors were 18.8 mm in height and 24 g in weight. Tactors had a diameter of 48.5 mm (Figure 1). The frequency of these two mastoid vibrations was set at 100 Hz and was controlled by software (TAction Creator, Engineering Acoustics, FL, United States). Applying this 100 Hz vibration has been proved to induce the slow-phase velocity of eye movement toward the vibrated side of mastoid process (Park et al., 2007). Vibration amplitudes were set at 130% of participants’ minimum perceived amplitudes (Lu et al., 2022). Participants were instructed to stand still while an experimenter adjusted the vibration amplitude through the commercial software TAction Creator until they were able to perceive the minimum perceived amplitude. It was an impulse type of vibration activation, indicating an activation period of 0.5 s and a deactivation period of 0.5 s. A purpose of using this type of impulse vibration was to reduce vestibular saturation (Lu et al., 2022).

### 2.3 Experimental protocol

The data collection was performed at Clinical Movement Analysis Laboratory at the University of Nebraska Medical Center. Dr. Li Zheng as an exchanged scholar and Dr. Jung Hung Chien collected, processed, and analyzed the data. Before the data collection, each participant needed to fill the Mini Mental State Examination. If the individual’s score of Mini Mental State Examination below 23, the experiment was terminated. During one visit, a total of six standing trials (SOT1-N: normal standing on the fixed surface without any VS, SOT1-Bi: normal standing on the fixed surface with bilateral VS, SOT1-Uni: normal standing on the fixed surface with unilateral VS, SOT5-N: standing on sway-reference surface and was blindfolded without any VS, SOT5-Bi: standing on the sway-reference surface and was blindfolded with bilateral VS, and SOT5-Uni: standing one the sway-reference and was blindfolded with unilateral VS) were randomly provided to each participant. The randomization procedures were as follows. Firstly, conditions SOT1-N, SOT1-Bi, SOT1-Uni, SOT5-N, SOT5-Bi, and SOT5-Uni were labeled as conditions 1, 2, 3, 4, 5, and 6. Then, the RANDBETWEEN formula built in Microsoft Excel (Microsoft, Seattle, WA, United States) was a method to select a range of numbers (1–6) to use in the randomizing process. Each trial lasted for 90 s (Chien et al., 2014). Participants’ feet were placed according to the method described in Vanicek et al. (2013). Participants were instructed to stand upright on the two force plates (one for each foot) without shoes, and to place their feet according to their height. The ankles of the participant should be aligned with the thick horizontal line running through the axis of rotation of the force plate. This can be achieved by aligning the experimenter’s thumb with the participant’s medial malleolus and the experimenter’s fingers with the horizontal line. Following this, the participant should place the outside of their heels on the vertical line marked by the letter “T” on the surface of the force plate. Also, these participants were instructed to keep their arms relaxed at their sides throughout each data collection. To ensure consistency between participants, the unilateral VS was administered through the tactor placed on the right mastoid process. Between trials, a one-minute of mandatory sitting rest was assigned (Lin et al., 2022). After 1 min rest, participants were asked to perform sit-to-stand and to walk 6 m straightly. Then, a short questionnaire, Niigata Persistent Postural-Perceptual Dizziness questionnaire (Yagi et al., 2019) was administered to each participant at the end of each trial to assess whether they experienced any uncomfortable sensations after this standing trial. The question #Q1, #Q3, #Q6, and #Q7 were selected in this questionnaire as follows: Q1) Quick movements such as standing up or turning your head, Q3) Walking at a natural pace, Q6) Sitting upright in a seat without back and arm support, and Q7) standing without touching fixed objects. Participants were asked “please indicate your answer by circling a Yes or No that best describe the extent to which you feel any discomfort or dizziness during or after the experimental trial.” The experiment was terminated if the answer from this questionnaire was filled as “Yes,” or participants felt any discomfort during any of the standing conditions. A data collection took approximately one and a half hours.

### 2.4 Data analysis

Three types of measures in the present study were used to evaluate the balance control as follows: The total traveling distance (Lemay et al., 2014), Performance Ratio (Nashner et al., 1982; Chien et al., 2014), and Sample Entropy (Montesinos et al., 2018).

#### 2.4.1 Total traveling distance of CoG

A total traveling distance was the sum of CoG moving distance between two time points (e.g., the CoG distance from the zero to the 0.01 s, and from the 0.01 s to the 0.02 s and so on). The total traveling distance of CoG was calculated in both AP and ML directions.

$$Total\;Traveling\;Distance\;of\;CoG=\sum_{time=0s}^{time=90s}\left|{CoG}_{time+0.01s}-{CoG}_{time}\right|$$

#### 2.4.2 Performance ratio of CoG (PR)

Using the numerical integral of the rectified sway signal (removed from the steady-state offset) and scaling the results as a percentage of maximum sway during standing, a PR was determined (Nashner et al., 1982). Before data collection was given, participants need to stand on force plates with eye-opened and fixed surface for 10 s. The CoG coordinates in AP and ML directions in these 10 s were averaged as the CoGsteady_state. Then, participants were instructed to lean forward, backward, lateral-toward-right or lateral-toward-left as possible as they can before taking a step. Therefore, CoGmax in the AP direction was defined as range of the maximum lean forward (anterior direction) and the maximum lean backward (posterior direction) before taking a step. Also, CoGmax in the ML direction was defined as range of the maximum lateral-toward-right and the maximum lateral-toward-left before taking a step. PR values approaching 100 indicate a loss of balance, whereas PR values approaching zero indicate stable postural control. Also, if participants stepped off from the force plates, PR values was assigned as 100 immediately.

$$\mathrm{PR}=\sum_{time=0s}^{time=90s}\frac{\left|{CoG}_{time}-{CoG}_{steady\_state}\right|}{{CoG}_{\max}-{CoG}_{steady\_state}}$$

#### 2.4.3 Sample entropy (SampEn) of CoG

The sample entropy was used to assess the regularity or predictability within CoG movements in time-series collected under different conditions and in different experimental groups (e.g., genders). The working definition in studies (Richman and Mooram, 2000; Lubetzky et al., 2018; Montesinos et al., 2018; Lin et al., 2022) was that more regular movements represented more predictable movements, required less degree of freedom of movements, and produced lower entropy values. In contrast, more irregular movements indicated less predictable movements, required higher degrees of freedom of movements, and produced higher entropy values. SampEn was calculated by the negative natural logarithm of the conditional properties that a series of data pointed a certain distance apart, *m*, would repeat itself at *m+1*. Also, SampEn took the logarithm of the sum of conditional probabilities as SampEn (*m*, γ, τ, *N*), where *m* was the embedding dimension, γ was tolerance, τ was the time delay, and *N* was a time-series data set of length.

$$SampEn=-\ln\frac{d\left[X_{m+1}\left(i\right),X_{m+1}\left(j\right)\right]}{d\left[X_{m}\left(i\right),X_{m}\left(j\right)\right]}$$

where both $d\left[x_{m}\left(i\right),x_{m}\left(j\right)\right]$ and $d\left[x_{m+1}\left(i\right),x_{m+1}\left(j\right)\right]$ were smaller than *γ*. The *γ* = 0.2 and *m* = 3 were set in this study, followed by Montesinos et al. (2018). In Montesinos et al.‘s study, applying *γ* = 0.2 and *m* = 3 can identify the subtle differences in center of pressure trajectories between the older fallers and older non-fallers. This present study also followed Lin et al., 2022 that applying time delay (τ = 5) to the CoG data in both AP and ML directions. After an adjustment from 100 Hz to 20 Hz using time delay (τ = 5), a length of *N* became a data set of 1,800 data points (20 Hz × 90 s; Lin et al., 2022; Montesinos et al., 2018). The rationale using time delay was that using a unity delay (τ = 1) might only catch the linear autocorrelation properties of the signal and would mask the ability of SampEn to quantify the “true” regularity and non-linear feature in the time-series (Kaffashi et al., 2008).

### 2.5 Statistical analysis

A Shapiro-Wilk normality test with an alpha value of 0.05 was used to evaluate the normality for each dependent variable. The dependent variables were total traveling distance of CoG, PR of CoG and SampEn of CoG values in both AP and ML directions. Also, an independent t-test was used to compare the weight and height between females and males.

• If the data were normally distributed, a three-way mixed repeated measure ANOVA (2 SOT conditions x 3 VS x 2 sex groups) was applied to investigate the VS effect, the sex effect, VS effect as well as the interaction between these three effects. Post-hoc comparisons using the Tukey method were performed if an interaction existed in each dependent variable.

• If the data was not normally distributed, the Brunner and Langer non-parametric longitudinal data model was used to investigate the within-participant effect (2 SOT conditions x 3 VS conditions) and the between-participants effect (2 sex groups) (Brunner et al., 2002). The Wilcoxon Signed Rank Test was used for *post hoc* comparisons comparing the effects of different SOT conditions in each group if there existed an interaction. Comparing sex groups in each SOT condition with/without different VS were conducted using the Mann-Whitney U-test.

The sample size of this study was based on the previous study’s result (Chiu and Wang, 2007), recruitment of 15 males and 15 females would generate a power of 80% and a level of significance of 5% (two-sided) for detecting a true difference in muscle activation between the males and females during walking. The partial eta squared method was used to evaluate effect size in the present study, based on Cohen’s guideline 0.138 for a large effect size, 0.059 for a moderate effect size, and 0.01 for a small effect size (Cohen, 1988). Statistical analysis was completed in SPSS 26.0 (IBM Corporation, Armond, NY).

## 3 Results

### 3.1 Participant’s information

In this study, there was no statistical sex differences in age (females: 35.06 ± 17.83 years old vs. males: 34.80 ± 17.50 years old) but in weight (females: 60.93 ± 9.31 kg vs. 75.4 ± 10.29 kg, *p* < 0.001) and in height (females: 165.60 ± 5.09 kg vs. 174.80 ± 6.00 kg, *p* < 0.001).

### 3.2 Normality tests

The results of the Shapiro-Wilk test revealed that the alpha value was greater than 0.05 for total traveling distances, PR values, SampEn values in both AP and ML directions, indicating the normal distribution. Thus, a three-way mixed ANOVA (2 SOT conditions x 3 VS x 2 sex groups) was applied to investigate the VS effect, the sex effect, VS effect as well as the interaction between these three effects.

### 3.3 The results of mixed three-way repeated measure

#### 3.3.1 The effect of sex

A significant effect of sex was found in the PR in the AP direction (F_1, 28_ = 5.548, *p* = 0.026), in the SampEn values in the AP directions (F_1, 28_ = 5.611, *p* = 0.025) and in the ML direction (F_1,28_ = 92.164, *p* < 0.001). The results showed that females demonstrated greater PR value in the AP direction than in males. Also, significantly greater Entropy values were found in both AP and ML directions. More details are shown in Tables 1–6.

**TABLE 1:** The statistical results of Performance ratio of Center of Gravity in the anterior-posterior direction (PR_AP). SOT1, sensory organization test condition 1; SOT5, sensory organization test condition 5; VS, vestibular stimulation; N, no VS, Uni, unilateral VS; Bi, bilateral VS; Female-SOT1, females stood in SOT1; Female-SOT5, females stood in SOT5; Male-SOT1, males stood in SOT1; Male-SOT5, males stood in SOT5; Female-N, female stood without VS; Female-Uni, females stood with unilateral VS; Female-Bi, females stood with bilateral VS; Male-N, males stood without VS; Male-Uni, males stood with unilateral VS; Male-Bi, males stood with bilateral VS; SOT1-N, standing in SOT1 without VS; SOT1-Uni, standing in SOT1 with unilateral VS; SOT1-Bi, standing in SOT1 with bilateral VS; SOT5-N, standing in SOT5 without VS; SOT5-Uni, standing in SOT5 with unilateral VS; SOT5-Bi, standing in SOT5 with bilateral VS; NA, the interaction did not reach the significant level; NS, not significant.

PR_AP (%)		Means (Std)	Sex	SOT Conditions	VS	Sex x SOT Conditions	Sex x VS	VS x SOT Conditions	Sex x SOT Conditions x VS					
SOT1-N	Female	13.432 (3.128)	*p* = 0.026	*p* < 0.001	*p* < 0.001	*p* = 0.022	*p* = 0.247	*p* < 0.001	*p* = 0.059					
	Male	13.350 (4.884)												
SOT1-Uni	Female	19.228 (5.792)	Sex x SOT Conditions	Female-SOT1	Male-SOT1	Female-SOT5	Male-SOT5	Sex x VS	Female-N	Female-Uni	Female-Bi	Male-N	Male-Uni	Male-Bi
	Male	17.594 (5.726)	Female-SOT1		NS	*p* < 0.001	*p* < 0.001	Female-N		NA	NA	NA	NA	NA
SOT1-Bi	Female	22.235 (6.293)	Male-SOT1	NS		*p* < 0.001	*p* < 0.001	Female-Uni	NA		NA	NA	NA	NA
	Male	20.808 (7.292)	Female-SOT5	*p* < 0.001	*p* < 0.001		*p* < 0.001	Female-Bi	NA	NA		NA	NA	NA
SOT5-N	Female	59.082 (23.543)	Male-SOT5	*p* < 0.001	*p* < 0.001	*p* < 0.001		Male-N	NA	NA	NA		NA	NA
	Male	40.425 (8.506)						Male-Uni	NA	NA	NA	NA		NA
SOT5-Uni	Female	64.323 (21.737)	Sex	Female vs. Male				Male-Bi	NA	NA	NA	NA	NA	
	Male	50.228 (11.709)		*p* = 0.026				VS x SOT Conditions	SOT1-N	SOT1-Uni	SOT1-Bi	SOT5-N	SOT5-Uni	SOT5-Bi
SOT5-Bi	Female	73.805 (18.324)	SOT Conditions	SOT1 vs. SOT5				SOT1-N		NS	NS	*p* < 0.001	*p* < 0.001	*p* < 0.001
	Male	61.789 (12.749)		*p* < 0.001				SOT1-Uni	NS		NS	*p* < 0.001	*p* < 0.001	*p* < 0.001
			VS	N vs. Uni	N vs. Bi	Uni Vs. Bi		SOT1-Bi	NS	NS		*p* < 0.001	*p* < 0.001	*p* < 0.001
				*p* < 0.001	*p* < 0.001	*p* < 0.001		SOT5-N	*p* < 0.001	*p* < 0.001	*p* < 0.001		NS	*p* < 0.001
								SOT5-Uni	*p* < 0.001	*p* < 0.001	*p* < 0.001	NS		*p* = 0.037
								SOT5-Bi	*p* < 0.001	*p* < 0.001	*p* < 0.001	*p* < 0.001	*p* = 0.037	

**TABLE 2:** The statistical results of Performance ratio of Center of Gravity in the medial-lateral direction (PR_ML). SOT1, sensory organization test condition 1; SOT5, sensory organization test condition 5; VS, vestibular stimulation; N, no VS; Uni, unilateral VS; Bi, bilateral VS; Female-SOT1, females stood in SOT1; Female-SOT5, females stood in SOT5; Male-SOT1, males stood in SOT1; Male-SOT5, males stood in SOT5; Female-N, female stood without VS; Female-Uni, females stood with unilateral VS; Female-Bi, females stood with bilateral VS; Male-N, males stood without VS; Male-Uni, males stood with unilateral VS; Male-Bi, males stood with bilateral VS; SOT1-N, standing in SOT1 without VS; SOT1-Uni, standing in SOT1 with unilateral VS; SOT1-Bi, standing in SOT1 with bilateral VS; SOT5-N, standing in SOT5 without VS; SOT5-Uni, standing in SOT5 with unilateral VS; SOT5-Bi, standing in SOT5 with bilateral VS; NA, the interaction did not reach the significant level; NS, not significant.

PR_ML (%)		Means (Std)	Sex	SOT conditions	VS	Sex x SOT conditions	Sex x VS	VS x SOT conditions	Sex x SOT conditions x VS					
SOT1-N	Female	13.578 (2.987)	*p* = 0.263	*p* < 0.001	*p* < 0.001	*p* = 0.015	*p* < 0.001	*p* = 0.003	*p* < 0.001					
	Male	14.836 (5.267)												
SOT1-Uni	Female	26.118 (9.505)	Sex x SOT Conditions	Female-SOT1	Male-SOT1	Female-SOT5	Male-SOT5	Sex x VS	Female-N	Female-Uni	Female-Bi	Male-N	Male-Uni	Male-Bi
	Male	25.882 (8.983)	Female-SOT1		NS	*p* < 0.001	*p* < 0.001	Female-N		*p* = 0.028	*p* = 0.009	NS	*p* = 0.009	NS
SOT1-Bi	Female	19.731 (8.163)	Male-SOT1	NS		*p* < 0.001	*p* < 0.001	Female-Uni	*p* = 0.028		NS	*p* = 0.001	NS	NS
	Male	20.754 (7.181)	Female-SOT5	*p* < 0.001	*p* < 0.001		*p* = 0.018	Female-Bi	*p* = 0.009	NS		*p* < 0.001	NS	NS
SOT5-N	Female	30.967 (7.705)	Male-SOT5	*p* < 0.001	*p* < 0.001	*p* = 0.018		Male-N	NS	*p* = 0.001	*p* < 0.001		*p* < 0.001	NS
	Male	23.591 (7.076)						Male-Uni	*p* = 0.009	NS	NS	*p* < 0.001		NS
SOT5-Uni	Female	39.282 (12.732)	Sex	Female vs. Male				Male-Bi	NS	NS	NS	NS	NS	
	Male	42.014 (15.693)		*p* = 0.263				VS x SOT Conditions	SOT1-N	SOT1-Uni	SOT1-Bi	SOT5-N	SOT5-Uni	SOT5-Bi
SOT5-Bi	Female	48.182 (15.872)	SOT Conditions	SOT1 vs. SOT5				SOT1-N		*p* < 0.001	NS	*p* < 0.001	*p* < 0.001	*p* < 0.001
	Male	30.939 (10.685)		*p* < 0.001				SOT1-Uni	*p* < 0.001		NS	NS	*p* < 0.001	*p* < 0.001
			VS	N vs. Uni	N vs. Bi	Uni Vs. Bi		SOT1-Bi	NS	NS		NS	*p* < 0.001	*p* < 0.001
				*p* < 0.001	*p* < 0.001	*p* = 0.011		SOT5-N	*p* < 0.001	NS	NS		*p* < 0.001	*p* < 0.001
								SOT5-Uni	*p* < 0.001	*p* < 0.001	*p* < 0.001	*p* < 0.001		NS
								SOT5-Bi	*p* < 0.001	*p* < 0.001	*p* < 0.001	*p* < 0.001	NS	

**TABLE 3:** The statistical results of Sample Entropy of Center of Gravity in the anterior-posterior direction (SampEn_AP). SOT1, sensory organization test condition 1; SOT5, sensory organization test condition 5; VS, vestibular stimulation; N, no VS; Uni, unilateral VS; Bi, bilateral VS; Female-SOT1, females stood in SOT1; Female-SOT5, females stood in SOT5; Male-SOT1, males stood in SOT1; Male-SOT5, males stood in SOT5; Female-N, female stood without VS; Female-Uni, females stood with unilateral VS; Female-Bi, females stood with bilateral VS; Male-N, males stood without VS; Male-Uni, males stood with unilateral VS; Male-Bi, males stood with bilateral VS; SOT1-N, standing in SOT1 without VS; SOT1-Uni, standing in SOT1 with unilateral VS; SOT1-Bi, standing in SOT1 with bilateral VS; SOT5-N, standing in SOT5 without VS; SOT5-Uni, standing in SOT5 with unilateral VS; SOT5-Bi, standing in SOT5 with bilateral VS; NA, the interaction did not reach the significant level; NS, not significant.

SampEn_AP		Means(Std)	Sex	SOT conditions	VS	Sex x SOT conditions	Sex x VS	VS x SOT conditions	Sex x SOT conditions x VS					
SOT1-N	Female	0.134 (0.040)	*p* = 0.025	*p* < 0.001	*p* < 0.001	*p* = 0.002	*p* = 0.012	*p* < 0.001	*p* = 0.002					
	Male	0.108 (0.036)												
SOT1-Uni	Female	0.164 (0.056)	Sex x SOT Conditions	Female-SOT1	Male-SOT1	Female-SOT5	Male-SOT5	Sex x VS	Female-N	Female-Uni	Female-Bi	Male-N	Male-Uni	Male-Bi
	Male	0.126 (0.033)	Female-SOT1		*p* < 0.001	*p* < 0.001	*p* < 0.001	Female-N		NS	NS	NS	NS	NS
SOT1-Bi	Female	0.207 (0.059)	Male-SOT1	*p* < 0.001		*p* < 0.001	*p* < 0.001	Female-Uni	NS		NS	NS	NS	NS
	Male	0.138 (0.037)	Female-SOT5	*p* < 0.001	*p* < 0.001		NS	Female-Bi	NS	NS		NS	NS	NS
SOT5-N	Female	0.074 (0.019)	Male-SOT5	*p* < 0.001	*p* < 0.001	NS		Male-N	NS	NS	NS		NS	NS
	Male	0.074 (0.022)						Male-Uni	NS	NS	NS	NS		NS
SOT5-Uni	Female	0.061 (0.019)	Sex	Female vs. Male				Male-Bi	NS	NS	NS	NS	NS	
	Male	0.063 (0.019)		*p* = 0.025				VS x SOT Conditions	SOT1-N	SOT1-Uni	SOT1-Bi	SOT5-N	SOT5-Uni	SOT5-Bi
SOT5-Bi	Female	0.051 (0.015)	SOT Conditions	SOT1 vs. SOT5				SOT1-N		NS	*p* < 0.001	*p* < 0.001	*p* < 0.001	*p* < 0.001
	Male	0.055 (0.018)		*p* < 0.001				SOT1-Uni	NS		NS	*p* < 0.001	*p* < 0.001	*p* < 0.001
			VS	N vs. Uni	N vs. Bi	Uni Vs. Bi		SOT1-Bi	*p* < 0.001	NS		*p* < 0.001	*p* < 0.001	*p* < 0.001
				*p* = 0.091	*p* = 0.023	*p* = 0.021		SOT5-N	*p* < 0.001	*p* < 0.001	*p* < 0.001		NS	NS
								SOT5-Uni	*p* < 0.001	*p* < 0.001	*p* < 0.001	NS		NS
								SOT5-Bi	*p* < 0.001	*p* < 0.001	*p* < 0.001	NS	NS	

**TABLE 4:** The statistical results of Sample Entropy of Center of Gravity in the medial-lateral direction (SampEn_ML). SOT1, sensory organization test condition 1; SOT5, sensory organization test condition 5; VS, vestibular stimulation; N, no VS; Uni, unilateral VS; Bi, bilateral VS; Female-SOT1, females stood in SOT1; Female-SOT5, females stood in SOT5; Male-SOT1, males stood in SOT1; Male-SOT5, males stood in SOT5; Female-N, female stood without VS; Female-Uni, females stood with unilateral VS; Female-Bi, females stood with bilateral VS; Male-N, males stood without VS; Male-Uni, males stood with unilateral VS; Male-Bi, males stood with bilateral VS; SOT1-N, standing in SOT1 without VS; SOT1-Uni, standing in SOT1 with unilateral VS; SOT1-Bi, standing in SOT1 with bilateral VS; SOT5-N, standing in SOT5 without VS; SOT5-Uni, standing in SOT5 with unilateral VS; SOT5-Bi, standing in SOT5 with bilateral VS; NA, the interaction did not reach the significant level; NS, not significant.

SampEn_ML		Means (Std)	Sex	SOT conditions		VS	Sex x SOT conditions	Sex x VS	VS x SOT conditions	Sex x SOT conditions x VS					
SOT1-N	Female	0.175 (0.049)	*p* < 0.001	*p* < 0.001	*p* < 0.001		*p* < 0.001	*p* < 0.001	*p* < 0.001	*p* < 0.001					
	Male	0.071 (0.023)													
SOT1-Uni	Female	0.349 (0.099)	Sex x SOT Conditions	Female-SOT1	Male-SOT1		Female-SOT5	Male-SOT5	Sex x VS	Female-N	Female-Uni	Female-Bi	Male-N	Male-Uni	Male-Bi
	Male	0.092 (0.028)	Female-SOT1		*p* < 0.001		*p* < 0.001	*p* < 0.001	Female-N		*p* = 0.001	NS	*p* < 0.001	*p* = 0.001	*p* = 0.001
SOT1-Bi	Female	0.247 (0.059)	Male-SOT1	*p* < 0.001			*p* = 0.001	NS	Female-Uni	*p* = 0.001		*p* = 0.003	*p* < 0.001	*p* < 0.001	*p* < 0.001
	Male	0.116 (0.047)	Female-SOT5	*p* < 0.001	*p* = 0.001			*p* < 0.001	Female-Bi	NS	*p* = 0.003		*p* < 0.001	*p* < 0.001	*p* < 0.001
SOT5-N	Female	0.174 (0.034)	Male-SOT5	*p* < 0.001	NS		*p* < 0.001		Male-N	*p* < 0.001	*p* < 0.001	*p* < 0.001		NS	NS
	Male	0.069 (0.018)							Male-Uni	*p* = 0.001	*p* < 0.001	*p* < 0.001	NS		NS
SOT5-Uni	Female	0.142 (0.031)	Sex	Female vs. Male					Male-Bi	*p* = 0.001	*p* < 0.001	*p* < 0.001	NS	NS	
	Male	0.114 (0.031)		*p* < 0.001					VS x SOT Conditions	SOT1-N	SOT1-Uni	SOT1-Bi	SOT5-N	SOT5-Uni	SOT5-Bi
SOT5-Bi	Female	0.112 (0.029)	SOT Conditions	SOT1 vs. SOT5					SOT1-N		*p* < 0.001	NS	NS	NS	NS
	Male	0.088 (0.018)		*p* < 0.001					SOT1-Uni	*p* < 0.001		NS	*p* < 0.001	*p* < 0.001	*p* < 0.001
			VS	N vs. Uni	N vs. Bi		Uni Vs. Bi		SOT1-Bi	NS	NS		NS	NS	*p* = 0.002
				*p* < 0.001	*p* < 0.001		*p* = 0.022		SOT5-N	NS	*p* < 0.001	NS		NS	NS
									SOT5-Uni	NS	*p* < 0.001	NS	NS		NS
									SOT5-Bi	NS	*p* < 0.001	*p* = 0.002	NS	NS	

**TABLE 5:** The statistical results of Traveling distance of Center of Gravity in the anterior-posterior direction (TD in AP). SOT1, sensory organization test condition 1; SOT5, sensory organization test condition 5; VS, vestibular stimulation; N, no VS, Uni, unilateral VS; Bi, bilateral VS; Female-SOT1, females stood in SOT1; Female-SOT5, females stood in SOT5; Male-SOT1, males stood in SOT1; Male-SOT5, males stood in SOT5; Female-N, female stood without VS; Female-Uni, females stood with unilateral VS; Female-Bi, females stood with bilateral VS; Male-N, males stood without VS; Male-Uni, males stood with unilateral VS; Male-Bi, males stood with bilateral VS; SOT1-N, standing in SOT1 without VS; SOT1-Uni, standing in SOT1 with unilateral VS; SOT1-Bi, standing in SOT1 with bilateral VS; SOT5-N, standing in SOT5 without VS; SOT5-Uni, standing in SOT5 with unilateral VS; SOT5-Bi, standing in SOT5 with bilateral VS; NS, not significant.

TD in AP (m)			Means (Std)	Sex	SOT conditions	VS	Sex x SOT conditions	Sex x VS		VS x SOT conditions	Sex x SOT conditions x VS					
SOT1-N	Female		1.396 (0.235)	*p* = 0.158	*p* < 0.001	*p* < 0.001	*p* = 0.024	*p* = 0.024		*p* < 0.001	*p* = 0.041					
	Male		1.203 (0.141)													
SOT1-Uni	Female		1.468 (0.232)	Sex x SOT Conditions	Female-SOT1	Male-SOT1	Female-SOT5	Male-SOT5		Sex x VS	Female-N	Female-Uni	Female-Bi	Male-N	Male-Uni	Male-Bi
	Male		1.321 (0.167)	Female-SOT1		NS	*p* < 0.001	*p* < 0.001		Female-N		NS	NS	NS	NS	*p* = 0.018
SOT1-Bi	Female		1.567 (0.324)	Male-SOT1	*p* = 0.855		*p* < 0.001	*p* < 0.001		Female-Uni	NS		NS	NS	NS	NS
	Male		1.404 (0.189)	Female-SOT5	*p* < 0.001	*p* < 0.001		*p* = 0.001		Female-Bi	NS	NS		NS	NS	NS
SOT5-N	Female		2.597 (0.521)	Male-SOT5	*p* < 0.001	*p* < 0.001	*p* = 0.001			Male-N	NS	NS	NS		NS	*p* = 0.036
	Male		2.977 (1.113)							Male-Uni	NS	NS	NS	NS		NS
SOT5-Uni	Female		3.136 (0.729)	Sex	Female vs. Male					Male-Bi	*p* = 0.018	NS	NS	*p* = 0.036	NS	
	Male		3.924 (1.596)		*p* = 0.158					VS x Conditions	SOT1-N	SOT1-Uni	SOT1-Bi	SOT5-N	SOT5-Uni	SOT5-Bi
SOT5-Bi	Female		3.814 (1.038)	SOT Conditions	SOT1 vs. SOT5					SOT1-N		NS	NS	*p* < 0.001	*p* < 0.001	*p* < 0.001
	Male		5.051 (1.863)		*p* < 0.001					SOT1-Uni	NS		NS	*p* < 0.001	*p* < 0.001	*p* < 0.001
				VS	N vs. Uni	N vs. Bi	Uni Vs. Bi			SOT1-Bi	NS	NS		*p* < 0.001	*p* < 0.001	*p* < 0.001
					*p* < 0.001	*p* < 0.001	*p* < 0.001			SOT5-N	*p* < 0.001	*p* < 0.001	*p* < 0.001		*p* = 0.027	*p* < 0.001
										SOT5-Uni	*p* < 0.001	*p* < 0.001	*p* < 0.001	*p* = 0.027		*p* = 0.003
										SOT5-Bi	*p* < 0.001	*p* < 0.001	*p* < 0.001	*p* < 0.001	*p* = 0.003	

**TABLE 6:** The statistical results of Traveling distance of Center of Gravity in the medial-lateral direction (TD in ML). SOT1, sensory organization test condition 1, SOT5, sensory organization test condition 5; VS, vestibular stimulation; N, no VS; Uni, unilateral VS; Bi, bilateral VS; Female-SOT1, females stood in SOT1; Female-SOT5, females stood in SOT5; Male-SOT1, males stood in SOT1; Male-SOT5, males stood in SOT5; Female-N, female stood without VS; Female-Uni, females stood with unilateral VS; Female-Bi, females stood with bilateral VS; Male-N, males stood without VS; Male-Uni, males stood with unilateral VS; Male-Bi, males stood with bilateral VS; SOT1-N, standing in SOT1 without VS; SOT1-Uni, standing in SOT1 with unilateral VS; SOT1-Bi, standing in SOT1 with bilateral VS; SOT5-N, standing in SOT5 without VS; SOT5-Uni, standing in SOT5 with unilateral VS; SOT5-Bi, standing in SOT5 with bilateral VS; NA: the interaction did not reach the significant level; NS, not significant.

TD in ML (m)		Means (Std)	Sex	Conditions	VS	Sex x conditions	Sex x VS	VS x conditions	Sex x conditions x VS					
SOT1-N	Female	1.005 (0.157)	*p* = 0.251	*p* < 0.001	*p* < 0.001	*p* = 0.137	*p* = 0.384	*p* = 0.016	*p* = 0.802					
	Male	0.829 (0.109)												
SOT1-Uni	Female	1.034 (0.178)	Sex x Conditions	Female-SOT1	Male-SOT1	Female-SOT5	Male-SOT5	Sex x VS	Female-N	Female-Uni	Female-Bi	Male-N	Male-Uni	Male-Bi
	Male	0.919 (0.191)	Female-SOT1		NA	NA	NA	Female-N		NA	NA	NA	NA	NA
SOT1-Bi	Female	1.056 (0.186)	Male-SOT1	NA		NA	NA	Female-Uni	NA		NA	NA	NA	NA
	Male	0.966 (0.216)	Female-SOT5	NA	NA		NA	Female-Bi	NA	NA		NA	NA	NA
SOT5-N	Female	1.186 (0.219)	Male-SOT5	NA	NA	NA		Male-N	NA	NA	NA		NA	NA
	Male	1.135 (0.293)						Male-Uni	NA	NA	NA	NA		NA
SOT5-Uni	Female	1.375 (0.262)	Sex	Female vs. Male				Male-Bi	NA	NA	NA	NA	NA	
	Male	1.371 (0.209)		NS				VS x Conditions	SOT1-N	SOT1-Uni	SOT1-Bi	SOT5-N	SOT5-Uni	SOT5-Bi
SOT5-Bi	Female	1.346 (0.329)	Conditions	SOT1 vs. SOT5				SOT1-N		NS	NS	*p* = 0.001	*p* < 0.001	*p* < 0.001
	Male	1.314 (0.291)		*p* < 0.001				SOT1-Uni	NS		NS	*p* = 0.026	*p* < 0.001	*p* < 0.001
			VS	N vs. Uni	N vs. Bi	Uni Vs. Bi		SOT1-Bi	NS	NS		NS	*p* < 0.001	*p* < 0.001
				*p* < 0.001	*p* < 0.001	NS		SOT5-N	*p* = 0.001	*p* = 0.026	NS		*p* = 0.006	*p* = 0.054
								SOT5-Uni	*p* < 0.001	*p* < 0.001	*p* < 0.001	*p* = 0.006		NS
								SOT5-Bi	*p* < 0.001	*p* < 0.001	*p* < 0.001	*p* = 0.054	NS	

#### 3.3.2 The interaction between the effect of sex and the effect of different SOT conditions

A significant interaction was found in the total traveling distance in the AP direction (F_1, 28_ = 5.735, *p* = 0.024), in the PR in the AP direction (F_1, 28_ = 5.876, *p* = 0.022), in the PR in the ML direction (F_1, 28_ = 6.712, *p* = 0.015), in the SampEn values in the AP directions (F_1, 28_ = 11.749, *p* = 0.002) and in the ML direction (F_2, 56_ = 52.621, *p* < 0.001). More details are shown in Tables 1–6.

#### 3.3.3 The interaction between the effect of sex and the effect of VS

A significant interaction was found in the total traveling distance in the AP direction (F_2, 56_ = 3.975, *p* = 0.024), in the PR in the ML direction (F_2, 56_ = 9.308, *p* < 0.001), in the SampEn values in the AP directions (F_2, 56_ = 4.781, *p* = 0.012) and in the ML direction (F_2, 56_ = 20.572, *p* < 0.001). More details are shown in Tables 1–6.

#### 3.3.4 The interaction among the effect of sex, the effect of SOT condition, and the effect of VS

**FIGURE 2:**
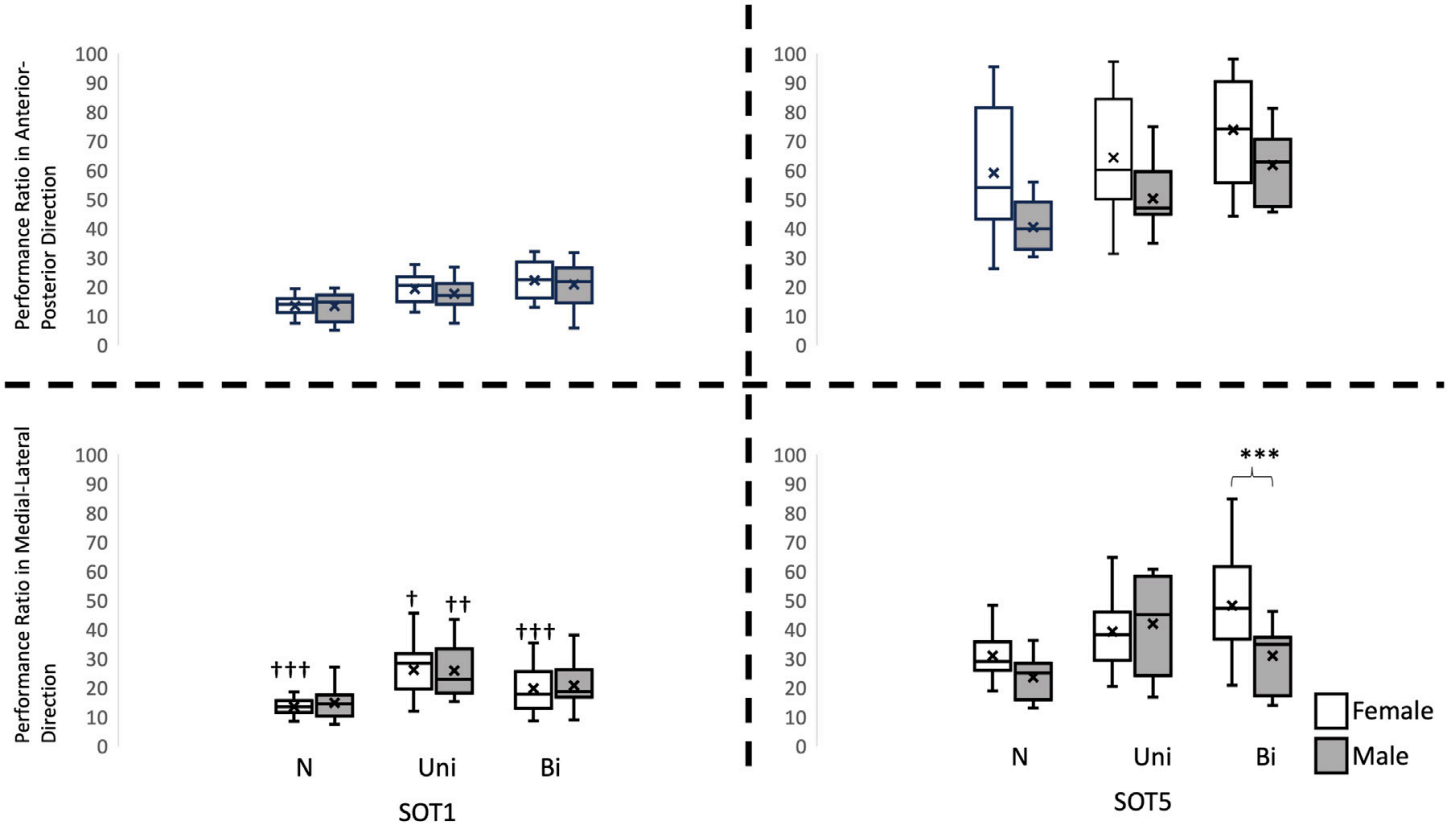
Performance ratio of Center of Gravity in the anterior-posterior and medial-lateral direction. †: indicates a significant difference between Sensory organization test condition 1 (SOT 1) and condition 5 (SOT 5). *: indicates a significant difference between genders. N: no vestibular stimulation. Uni: unilateral vestibular stimulation. Bi: bilateral vestibular stimulation.

**FIGURE 3:**
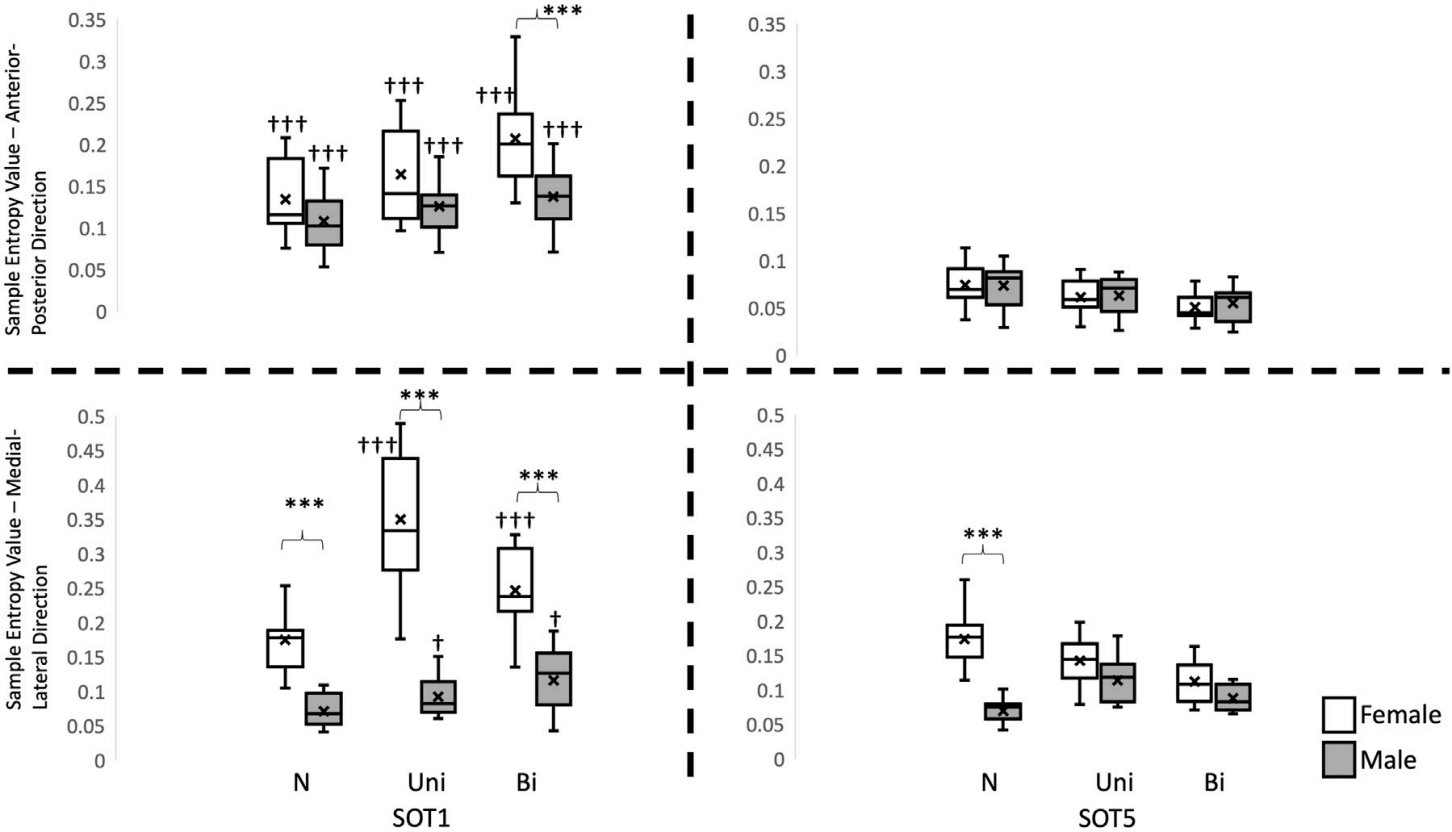
Sample Entropy values of Center of Gravity in the anterior-posterior and medial-lateral direction. †: indicates a significant difference between Sensory organization test condition 1 (SOT 1) and condition 5 (SOT 5). *: indicates a significant difference between genders. N: no vestibular stimulation. Uni: unilateral vestibular stimulation. Bi: bilateral vestibular stimulation.

**FIGURE 4:**
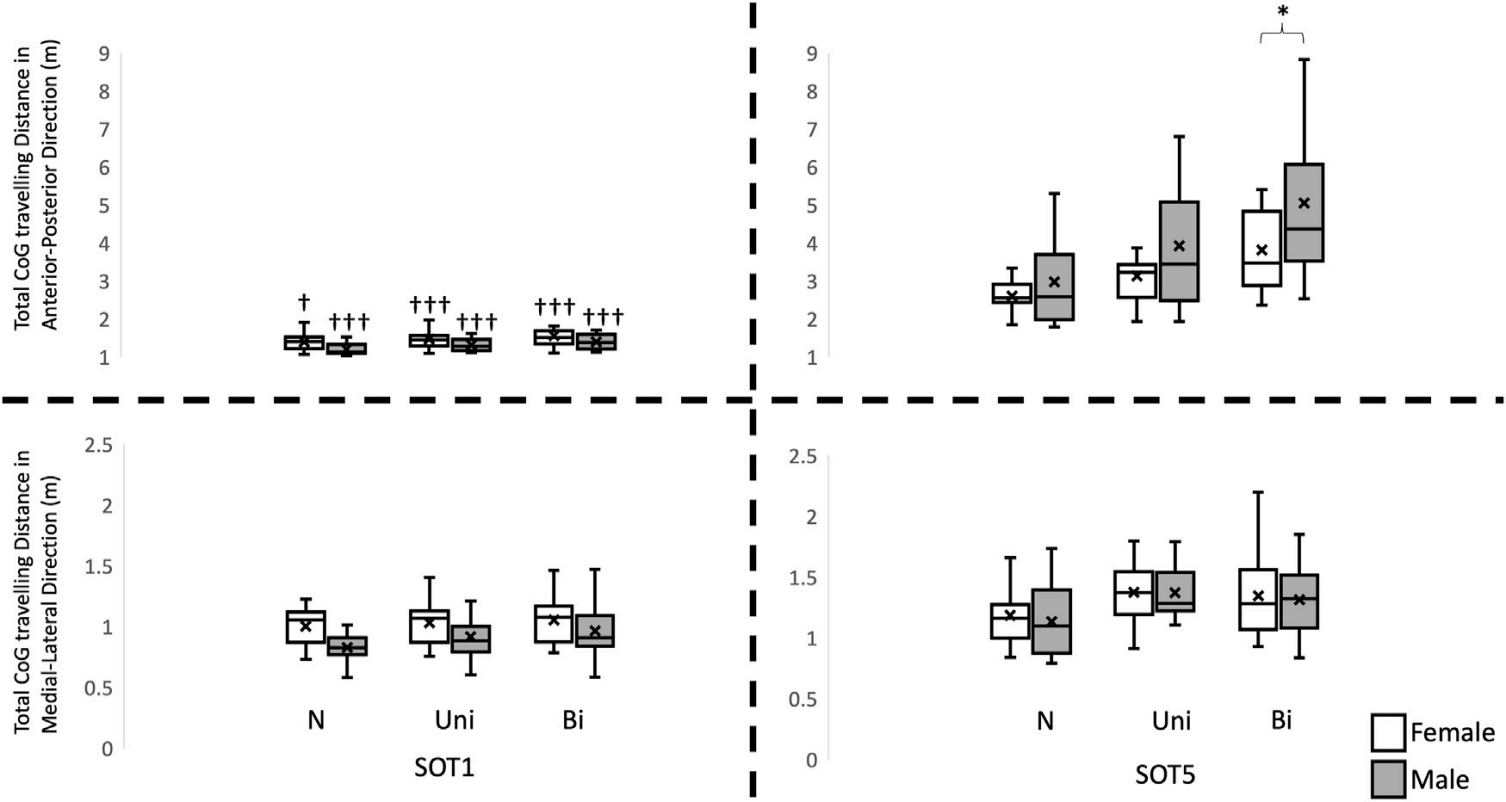
Total Center of gravity travelling distance in the anterior-posterior and medial-lateral direction. †: indicates a significant difference between Sensory organization test condition 1 (SOT 1) and condition 5 (SOT 5). *: indicates a significant difference between genders. N: no vestibular stimulation. Uni: unilateral vestibular stimulation. Bi: bilateral vestibular stimulation.

A significant interaction was found in total traveling distance in the AP direction (F_2, 56_ = 3.379, *p* = 0.041), in the PR in the ML direction (F_2, 56_ = 16.809, *p* < 0.001), in the SampEn values in the AP directions (F_2, 56_ = 7.118, *p* = 0.002) and in the ML direction (F_2, 56_ = 50.889, *p* < 0.001). Pairwise comparisons corrected by Tukey method revealed that the total traveling distance in the AP direction was significantly less in females than in males (*p* = 0.01, Figure 2), and the PR in the ML direction was significantly greater in females than in males while standing (*p* = 0.001, Figure 3) in SOT5 with Bi VS. Also, For the Entropy measure, significantly greater SampEn values were found in females than in males in the AP (*p* < 0.001) and in the ML (*p* < 0.001) directions while standing in SOT1 with Bi VS. More details were shown in Figure 4.

## 4 Discussions

It was the objective of this study to determine whether or not sex effect impacted balance control with/without VS during standing in normal and vestibular-demanding situations. Results mostly confirmed the hypothesis that 1) females demonstrated greater PR of CoG in the AP direction and SampEn of CoG in both AP and ML directions than males regardless of whether VS was administered or what SOT conditions participants were in, 2) All participants, regardless of whether VS was administered, showed an increase in PR of CoG, but a decrease in SampEn of CoG, when standing in SOT5 compared to standing in SOT1. Furthermore, females showed greater increases in PR and decreased SampEn of CoG than males in SOT5, 3) Regardless of the SOT conditions participants stood in, VS had a greater impact on females than on males, and 4) As compared to other conditions, females showed the greatest PR of CoG when standing in vestibular-demanding and bilaterally vestibular-disturbed conditions (SOT5, the most challenging task).

### 4.1 A sex effect on balance control

This study was funded by NASA in order to determine whether there were any differences in balance control between the sexes when performing a simple task - standing. Regardless of whether VS was administered or what SOT conditions participants were in, healthy females had a greater PR of CoG in the AP direction and a greater SampEn of CoG in both directions than healthy males. In contrast to males, females controlled balance by largely adjusting instantaneous sway, as evidenced by a greater PR of CoG. Moreover, a greater SampEn of CoG may be explained by the fact that females used higher degrees of freedom in their movement patterns (more irregular movements) as a result of an exploratory approach to maintain balance in both the AP and ML directions in time series (Chien et al., 2016). The above-mentioned differences in balance control between men and women may be the result of physiological and anatomical differences (Bowman et al., 2000; Corazzi et al., 2020; Lien and Yang, 2021; El Khiati et al., 2022), as well as differences in body mass and height (Greve et al., 2013). In light of these findings, it is apparent that both rehabilitation for patients with vestibular disorders and the diagnosis of astronauts’ sensorimotor functions should consider sex effects on balance control. It was also noted that the balance control could be improved by utilizing a real-time visual feedback system of CoG trajectory (Wang et al., 2021). Specifically, this type of training has been shown to improve balance control more in females than in males (Wang et al., 2021).

### 4.2 The interaction between the sex effect and SOT condition effect

As expected, both sexes showed a greater PR of CoG when standing under vestibular-demanding conditions (SOT5) than when standing normally (SOT1). Also, whether or not VS was applied, the sex effect on PR of CoG in both AP and ML directions as well as SampEn of CoG in ML directions was significant while standing SOT5. These results could be explained by the concept of internal model (Merfeld et al., 1999). The term internal model referred to the notion that the central nervous system (CNS) was capable of storing and updating information about the body in light of its external environment. To illustrate, in this study, a participant with blindfolded while standing on a sway-reference support surface (SOT5) could generate an initial internal mode (self-estimate) based on previous life experiences. As the surface began to rotate with respect to this participant, the initial internal model might become inaccurate. This might result in CNS having to prepare for another revision, thereby causing an increase in PR of CoG and an decrease in SampEn of CoG. It was possible that the PR of CoG of this participant might decrease gradually after becoming familiar with the scenario. Based on the abovementioned procedures, Ito (2008) proposed a conceptual structure of an internal model that control contained several major components: an instructor (prefrontal cortex), controller (motor cortex), controlled object (a body part), sensory system (visual, somatosensory, vestibular systems), forward model, and inverse model. In the current study, while standing in SOT5, the instructor (prefrontal cortex) first received environmental information from sensory systems (mostly from the vestibular system since two other sensory systems were disrupted) and then instructed the controller (motor cortex) accordingly. As a next step, the controller sent a motor command to a body part in order to maintain balance. Also, the controller sent a signal back to the internal model (forward model) to compare the actual body position with the predicted body position. When the predicted body position differs from the actual body position, the forward model may correct the differences and send the correction back to the motor cortex. As a result of repeatedly executing the procedure described above, the internal model was well-trained and turned to an inverse model for handling the similar situation rapidly next time, presenting better balance control in the same SOT 5 (Wolfson et al., 1994). As a result of this concept, it was reasonable to assume that the different anatomical structures within the vestibular system between males and females (Bowman et al., 2000; Corazzi et al., 2020; Lien and Yang, 2021; El Khiati et al., 2022) resulted in instructors (prefrontal cortex) giving different instructions to controllers (motor cortex) when the vestibular system was heavily relied. This resulted in a increase in PR of CoG and SampEn of CoG for females than males (when standing in SOT5). Despite the absence of sensorimotor training in this study, the above-mentioned procedure can serve as the basis for sensorimotor training in pathological groups, such as patients with vestibular disorders and astronauts. It was possible to develop an appropriate internal model for dealing with unfamiliar and unpredictable situations, such as those encountered on the moon and on Mars, by continuously exploring unfamiliar sensory-conflicted environment.

Standing in different sensory-conflicted situations, the PR of CoG would represent the outcomes of balance control, while the SampEn of CoG would represent the pathways to achieve these outcomes. One well-known example was Bernstein’s hammer stroke experiment (1923). A simple hammer stroke task could be extremely complex in Bernstein’s study (1923), and nails could be hit in a variety of ways, including abductive and vertical strokes. Despite similar trajectories, there were differences in the changes in joint coordinates or muscle activation over time in hammerheads. According to the findings of the present study, both males and females utilized lower degrees of freedom in balance control (less random) in SOT5 than in SOT1 in the AP direction. Furthermore, only females, but not males, exhibited a lower degree of freedom in balance control in the ML direction in SOT5 compared to SOT1. These decreases in degrees of freedom in males and females could be explained as the first response to handle unfamiliar situation. In the current study, none of these participants experienced standing in SOT5 environment; therefore, to limit the degree of freedom in the movement might be the most convenient method to maintain the balance control (Lin et al., 2022) while standing with blindfolded and sway-reference surface. It has been reported that females tended to rely on somatosensory system more than males to control balance while standing on moving surface (Schulleri et al., 2022). Thus, in SOT5, females reduced degrees of freedom not only in the AP direction, but also in the ML direction. This may be due to females having difficulties dealing with balance control while both visual and somatosensory systems were perturbed simultaneously. Importantly, the degree of freedom in both males and females in the present study could be used as a fundamental reference for identifying the progress in motor leaning (Guimarães et al., 2020) in pathological groups, such as patients with vestibular disorders or in astronauts in the future.

### 4.3 The interaction between the sex effect and VS effect

As far as we are aware, this was the first study to investigate the interaction between the sex effect and the VS effect on balance control regardless of whether the participants were standing in SOT1 or SOT5. It is interesting to note that females increased PR of CoG in the ML direction regardless of whether unilateral or bilateral VS was applied. In contrast, males increased PR of CoG in the ML direction only when unilateral VS was applied. Furthermore, only unilateral VS increased the SampEn of CoG in the ML direction in females whereas none of the VS affected the SampEn of CoG in males. Three observations need to be addressed with regard to these findings: 1) why did VS only affect balance control in the ML direction, but not in the AP direction, 2) why did VS have a greater effect on balance control in females than in males, 3) why did unilateral VS appear to affect SampEn of CoG more than bilateral VS in females than in males? Baudy and Kuo (2000) explain the first observation by the fact that balance control was more difficult in the ML direction than in the AP direction. It was possible that the second observation can be substantiated by the fact that females were more sensitive to vestibular signals than males (Sung et al., 2011; Battersby, 2019; Schubert et al., 2022). Third, despite standing normally with visual support, unilateral VS, but not bilateral VS, significantly increased the SampEn of CoG in the ML direction in older adults. In Lin et al., 2022 explanation, the deterioration of the vestibular system by aging caused older healthy adults to compensate for the disruption in the unilateral vestibular system by increasing their degree of freedom in the ML direction. As shown in the present study, it is not necessary to explain the phenomenon by a deterioration of vestibular function, rather, the higher sensitivity of vestibular system in females than in males was more likely to be the cause. These results confirmed the feasibility to use the VS to identify the differences in balance control between males and females, which has been used to identify the differences in balance control by aging (Lin et al., 2022). Therefore, it might be possible to apply this VS technology to identify the vestibular-related balance control in patients with various types of vestibular disorders and astronauts.

### 4.4 The interaction among SOT condition effect, sex effect and VS effect

When it comes to discussing the interaction between SOT condition effect, sex effect and VS effect, it has been found that PR of CoG in the ML and total CoG traveling distance in the AP direction were significantly different between males and females when standing in the most challenging condition (SOT5) with bilateral VS. Specifically, males demonstrated a greater total CoG traveling distance in the AP direction than females; however, females showed a greater PR of CoG in the ML direction than males in such a challenging condition. It is important to note that the total CoG traveling distance and the PR of CoG have been interpreted differently. The total CoG traveling distance represented the total amount distance the CoG travels; however, the PR of CoG indicated the sum of amplitude of instantaneous CoG travelled compared to the maximum amplitude of CoG travelled. On one hand, the greater body mass and height of males, according to Greve et al.'s (2012) study, explain greater total body travel distance than that of females while standing a such challenging condition. On the other hand, it must be taken into consideration that the greater amplitude of the instantaneous CoG traveled in a vestibular-disturbed and vestibular-demanding environment might increase the potential risks of falls for females.

### 4.5 Conclusion

The sex effect on vestibular function in astronauts generally has been ignored because most astronauts are males. As NASA intends to send a female to the Moon in 2024, it is essential to clarify how women differ from men when it comes to balance control while standing in a vestibular-demanding and unpredictable environment. As a result of the present study, it can be concluded that in the future, when performing space missions or evaluating vestibular function pre- and post-spaceflight, the sex effect must be taken into consideration. In addition, one strength of this study was the use of the VS as an unpredictable vestibular disruption in order to assess the sex differences in standing on an environment that is vestibular-demanding. It was also a strength of this study that multiple practical approaches were employed in order to quantify balance control in sensory-conflicted conditions. These results could serve as fundamental references for future comparisons of balance control between pathological groups and astronauts.

### 4.6 Limitations

There were a couple of limitations in the present study and need to be performed in the future:

• The purpose of the present study was to examine the sex differences in vestibular-related balance control while standing, rather than when walking, where frequent falls are more likely to occur. It is necessary to conduct further studies to examine the sex effect on vestibular-related balance control when walking in vestibular-demanding and vestibular-disrupted environments.
• Considering that no sensorimotor training was conducted in the present study, it is unknown whether training in such vestibular-demanding and vestibular-disrupted conditions would enhance the ability to maintain balance control in unpredictable environments. This question needs to be addressed in future studies.

## Data Availability

The original contributions presented in the study are included in the article/Supplementary Material, further inquiries can be directed to the corresponding authors.

## Abbreviations

SOT, sensory organization test; CoG, center of gravity; PR, Performance ratio; SampEn, Sample Entropy; AP, anterior-posterior; ML, medial-lateral; VS, vestibular stimulation; N, no vestibular stimulation; Uni, unilateral vestibular stimulation; Bi, bilateral vestibular stimulation.

## References

[B1] Battersby M. R. (2019). The effects of noise exposure on the peripheral vestibular system: an investigation of sex differences and threshold shift following vestibular damage. Senior Independent Study Theses. Paper 8371. Available at: https://openworks.wooster.edu/independentstudy/8371/ .

[B2] Bauby C. E. Kuo A. D. (2000). Active control of lateral balance in human walking. J. biomechanics 33 (11), 1433–1440. 10.1016/s0021-9290(00)00101-9 10940402

[B3] Bernstein N. A. (1923). Issledovania po biomekhanike udara s pomoshiu svetovoi zapisi (Studies of biomechanics of the strike with the camera recording) [in Russian]. Issledovanija Centr. Instituta Tr. 1, 19–79.

[B4] Black F. O. Shupert C. L. Peterka R. J. Nashner L. M. (1989). Effects of unilateral loss of vestibular function on the vestibulo-ocular reflex and postural control. Ann. otology, rhinology, laryngology 98 (11), 884–889. 10.1177/000348948909801109 2817680

[B5] Blazkiewicz M. Kędziorek J. Hadamus A. (2021). The impact of visual input and support area manipulation on postural control in subjects after osteoporotic vertebral fracture. Entropy (Basel, Switz. 23 (3), 375. 10.3390/e23030375 PMC8004071 33804770

[B60] Brunner E. Munzel U. Puri M. L. (2002). The multivariate nonparametric Behrens-Fisher problem. Journal of Statistical Planning and Inference 108, 37–53.

[B6] Bowman D. M. Brown D. K. Kimberley B. P. (2000). An examination of gender differences in DPOAE phase delay measurements in normal-hearing human adults. Hear. Res. 142 (1-2), 1–11. 10.1016/s0378-5955(99)00212-9 10748323

[B7] Chen W. Jiang F. Chen X. Feng Y. Miao J. Chen S. (2021). Photoplethysmography-derived approximate entropy and sample entropy as measures of analgesia depth during propofol-remifentanil anesthesia. J. Clin. Monit. Comput. 35 (2), 297–305. 10.1007/s10877-020-00470-6 32026257

[B8] Chien J. H. Eikema D. J. Mukherjee M. Stergiou N. (2014). Locomotor sensory organization test: a novel paradigm for the assessment of sensory contributions in gait. Ann. Biomed. Eng. 42 (12), 2512–2523. 10.1007/s10439-014-1112-7 25224076 PMC4241158

[B9] Chien J. H. Mukherjee M. Siu K. C. Stergiou N. (2016). Locomotor sensory organization test: how sensory conflict affects the temporal structure of sway variability during gait. Ann. Biomed. Eng. 44 (5), 1625–1635. 10.1007/s10439-015-1440-2 26329924 PMC4775456

[B10] Chiu M. C. Wang M. J. (2007). The effect of gait speed and gender on perceived exertion, muscle activity, joint motion of lower extremity, ground reaction force and heart rate during normal walking. Gait posture 25 (3), 385–392. 10.1016/j.gaitpost.2006.05.008 16814548

[B11] Cohen J. (1988). Statistical power analysis for the behavior sciences. 2nd ed. Lawrence Erlbaum Associate.

[B12] Corazzi V. Ciorba A. Skarżyński P. H. Skarżyńska M. B. Bianchini C. Stomeo F. (2020). Gender differences in audio-vestibular disorders. Int. J. Immunopathol. Pharmacol. 34, 2058738420929174. 10.1177/2058738420929174 32525749 PMC7290256

[B13] Di Fabio R. P. (1995). Sensitivity and specificity of platform posturography for identifying patients with vestibular dysfunction. Phys. Ther. 75 (4), 290–305. 10.1093/ptj/75.4.290 7899487

[B14] Donker S. F. Roerdink M. Greven A. J. Beek P. J. (2007). Regularity of center-of-pressure trajectories depends on the amount of attention invested in postural control. Exp. Brain Res. 181 (1), 1–11. 10.1007/s00221-007-0905-4 17401553 PMC1914290

[B15] El Khiati R. Tighilet B. Besnard S. Chabbert C. (2022). Hormones and vestibular disorders: the quest for biomarkers. Brain Sci. 12 (5), 592. 10.3390/brainsci12050592 35624978 PMC9139641

[B16] Faraldo-García A. Santos-Pérez S. Labella-Caballero T. Soto-Varela A. (2011). Influence of gender on the sensory organisation test and the limits of stability in healthy subjects. Acta otorrinolaringol. espanola 62 (5), 333–338. 10.1016/j.otorri.2011.03.003 21531358

[B17] Fischer O. M. Missen K. J. Tokuno C. D. Carpenter M. G. Adkin A. L. (2023). Postural threat increases sample entropy of postural control. Front. neurology 14, 1179237. 10.3389/fneur.2023.1179237 PMC10277644 37342783

[B18] Ford-Smith C. D. Wyman J. F. Elswick R. K. Fernandez T. Newton R. A. (1995). Test-retest reliability of the sensory organization test in noninstitutionalized older adults. Archives Phys. Med. rehabilitation 76 (1), 77–81. 10.1016/s0003-9993(95)80047-6 7811180

[B19] Foreman M. D. Fletcher K. Mion L. C. Simon L. (1996). Assessing cognitive function. Geriatr. Nurs. (New York, N.Y.) 17 (5), 228–232. 10.1016/s0197-4572(96)80210-2 8924123

[B20] Goebel J. A. Paige G. D. (1989). Dynamic posturography and caloric test results in patients with and without vertigo. Otolaryngology--head neck Surg. official J. Am. Acad. Otolaryngology-Head Neck Surg. 100 (6), 553–558. 10.1177/019459988910000605 2501729

[B21] Greve J. M. Cuğ M. Dülgeroğlu D. Brech G. C. Alonso A. C. (2013). Relationship between anthropometric factors, gender, and balance under unstable conditions in young adults. BioMed Res. Int. 2013, 850424. 10.1155/2013/850424 23509788 PMC3581282

[B22] Guimarães A. N. Ugrinowitsch H. Dascal J. B. Porto A. B. Okazaki V. H. A. (2020). Freezing degrees of freedom during motor learning: a systematic review. Mot. control 24 (3), 457–471. 10.1123/mc.2019-0060 32221040

[B23] Hamid M. A. Hughes G. B. Kinney S. E. (1991). Specificity and sensitivity of dynamic posturography. A retrospective analysis. Acta oto-laryngologica. Suppl. 481, 596–600. 10.3109/00016489109131480 1927480

[B24] Hansen C. Wei Q. Shieh J. S. Fourcade P. Isableu B. Majed L. (2017). Sample entropy, univariate, and multivariate multi-scale entropy in comparison with classical postural sway parameters in young healthy adults. Front. Hum. Neurosci. 11, 206. 10.3389/fnhum.2017.00206 28491029 PMC5405138

[B25] Horak F. B. Shupert C. L. Mirka A. (1989). Components of postural dyscontrol in the elderly: a review. Neurobiol. aging 10 (6), 727–738. 10.1016/0197-4580(89)90010-9 2697808

[B26] Hytönen M. Pyykkö I. Aalto H. Juhola M. Ramsay H. (1989). Vestibulo-ocular and vestibulo-spinal reflexes in evaluation of vestibular lesions. Acta oto-laryngologica. Suppl. 468, 231–234. 10.3109/00016488909139052 2635510

[B27] Ito M. (2008). Control of mental activities by internal models in the cerebellum. Nat. Rev. Neurosci. 9 (4), 304–313. 10.1038/nrn2332 18319727

[B28] Jain V. Wood S. J. Feiveson A. H. Black F. O. Paloski W. H. (2010). Diagnostic accuracy of dynamic posturography testing after short-duration spaceflight. Aviat. space, Environ. Med. 81 (7), 625–631. 10.3357/asem.2710.2010 20597240

[B29] Jia Y. Gu H. Luo Q. (2017). Sample entropy reveals an age-related reduction in the complexity of dynamic brain. Sci. Rep. 7 (1), 7990. 10.1038/s41598-017-08565-y 28801672 PMC5554148

[B30] Kaffasi F. Foglyano R. Wilson C. G. Lopara K. A. (2008). The effect of time delay on Approximate & Sample Entropy calculations. Phys. D. Nonlinear Phenom. 237 (23), 3069–3074. 10.1016/j.physd.2008.06.005

[B31] Kavounoudias A. Gilhodes J. C. Roll R. Roll J. P. (1999). From balance regulation to body orientation: two goals for muscle proprioceptive information processing? Exp. Brain Res. 124 (1), 80–88. 10.1007/s002210050602 9928792

[B32] Lemay J. F. Gagnon D. H. Nadeau S. Grangeon M. Gauthier C. Duclos C. (2014). Center-of-pressure total trajectory length is a complementary measure to maximum excursion to better differentiate multidirectional standing limits of stability between individuals with incomplete spinal cord injury and able-bodied individuals. J. neuroengineering rehabilitation 11, 8. 10.1186/1743-0003-11-8 PMC3899383 24438202

[B33] Lien K. H. Yang C. H. (2021). Sex differences in the triad of acquired sensorineural hearing loss. Int. J. Mol. Sci. 22 (15), 8111. 10.3390/ijms22158111 34360877 PMC8348369

[B34] Lin Y. Mukherjee M. Stergiou N. Chien J. H. (2022). Using mastoid vibration to detect age-related uni/bilateral vestibular deterioration during standing. J. Vestib. Res. Equilib. Orientat. 32 (2), 145–154. 10.3233/VES-210042 34180442

[B35] Lu J. Xie H. Chien J. H. (2022). Different types of mastoid process vibrations affect dynamic margin of stability differently. Front. Hum. Neurosci. 16, 896221. 10.3389/fnhum.2022.896221 35832875 PMC9271872

[B36] Lubetzky A. V. Harel D. Lubetzky E. (2018). On the effects of signal processing on sample entropy for postural control. PloS one 13 (3), e0193460. 10.1371/journal.pone.0193460 29494625 PMC5832259

[B37] Martines F. Giustino V. Dispenza F. Galletti F. Rizzo S. Salvago P. (2021). “Body balance and postural control in patients with dizziness,” in Dizziness: prevalence, risk factors and management. Editors Martines F. Salvago P. (Nova Science Publishers, Inc), 173–193. Available at: https://iris.unipa.it/handle/10447/529562 .

[B38] Merfeld D. M. Zupan L. Peterka R. J. (1999). Humans use internal models to estimate gravity and linear acceleration. Nature 398 (6728), 615–618. 10.1038/19303 10217143

[B39] Messina G. Glustino V. Corrao A. Rizzo S. Salvago P. Martine F. (2021). “Postural disorders in patients with dizziness: from postural analysis to vestibular rehabilitation programs,” in Dizziness: prevalence, risk factors and management. Editors Martines F. Salvago P. (Nova Science Publishers, Inc), 229–245. Available at: https://iris.unipa.it/handle/10447/579430 .

[B40] Mishra A. Davis S. Speers R. Shepard N. T. (2009). Head shake computerized dynamic posturography in peripheral vestibular lesions. Am. J. audiology 18 (1), 53–59. 10.1044/1059-0889(2009/06-0024 19307290

[B41] Montesinos L. Castaldo R. Pecchia L. (2018). On the use of approximate entropy and sample entropy with centre of pressure time-series. J. neuroengineering rehabilitation 15 (1), 116. 10.1186/s12984-018-0465-9 PMC6291990 30541587

[B42] Mulavara A. P. Cohen H. S. Peters B. T. Sangi-Haghpeykar H. Bloomberg J. J. (2013). New analyses of the sensory organization test compared to the clinical test of sensory integration and balance in patients with benign paroxysmal positional vertigo. Laryngoscope 123 (9), 2276–2280. 10.1002/lary.24075 23553110 PMC3771405

[B43] Nashner L. M. Black F. O. Wall C. 3rd (1982). Adaptation to altered support and visual conditions during stance: patients with vestibular deficits. J. Neurosci. official J. Soc. Neurosci. 2 (5), 536–544. 10.1523/JNEUROSCI.02-05-00536.1982 PMC6564270 6978930

[B44] Nashner L. M. Peters J. F. (1990). Dynamic posturography in the diagnosis and management of dizziness and balance disorders. Neurol. Clin. 8 (2), 331–349. 10.1016/s0733-8619(18)30359-1 2193215

[B45] Ozdemir R. A. Goel R. Reschke M. F. Wood S. J. Paloski W. H. (2018). Critical role of somatosensation in postural control following spaceflight: vestibularly deficient astronauts are not able to maintain upright stance during compromised somatosensation. Front. physiology 9, 1680. 10.3389/fphys.2018.01680 PMC6277541 30538640

[B46] Park H. Shin J. Shim D. (2007). Mechanisms of vibration-induced nystagmus in normal subjects and patients with vestibular neuritis. Audiology neuro-otology 12 (3), 189–197. 10.1159/000099023 17259708

[B47] Richman J. S. Lake D. E. Moorman J. R. (2004). Sample entropy. Methods Enzym. 384, 172–184. 10.1016/S0076-6879(04)84011-4 15081687

[B48] Richman J. S. Moorman J. R. (2000). Physiological time-series analysis using approximate entropy and sample entropy. Am. J. physiology. Heart circulatory physiology 278 (6), H2039–H2049. 10.1152/ajpheart.2000.278.6.H2039 10843903

[B49] Schubert N. M. A. Roelofs C. G. Free R. H. Wiersinga-Post J. E. C. Pyott S. J. (2022). Age-related high-frequency hearing loss is not associated with horizontal semicircular canal function. Ear Hear. 43 (6), 1845–1852. 10.1097/AUD.0000000000001252 35696183 PMC9592157

[B50] Schulleri K. H. Johannsen L. Michel Y. Lee D. (2022). Sex differences in the association of postural control with indirect measures of body representations. Sci. Rep. 12 (1), 4556. 10.1038/s41598-022-07738-8 35296686 PMC8927351

[B51] Shishkin N. Kitov V. Sayenko D. Tomilovskaya E. (2023). Sensory organization of postural control after long term space flight. Front. neural circuits 17, 1135434. 10.3389/fncir.2023.1135434 37139078 PMC10149828

[B52] Sung P. H. Cheng P. W. Young Y. H. (2011). Effect of gender on ocular vestibular-evoked myogenic potentials via various stimulation modes. Clin. neurophysiology official J. Int. Fed. Clin. Neurophysiology 122 (1), 183–187. 10.1016/j.clinph.2010.06.004 20591729

[B53] Tays G. D. Hupfeld K. E. McGregor H. R. Salazar A. P. De Dios Y. E. Beltran N. E. (2021). The effects of long duration spaceflight on sensorimotor control and cognition. Front. neural circuits 15, 723504. 10.3389/fncir.2021.723504 34764856 PMC8577506

[B54] Vanicek N. King S. A. Gohil R. Chetter I. C. Coughlin P. A. (2013). Computerized dynamic posturography for postural control assessment in patients with intermittent claudication. J. Vis. Exp. JoVE (82), e51077. 10.3791/51077 24378378 PMC4047968

[B55] Vereeck L. Wuyts F. Truijen S. Van de Heyning P. (2008). Clinical assessment of balance: normative data, and gender and age effects. Int. J. audiology 47 (2), 67–75. 10.1080/14992020701689688 18236239

[B56] Wang I. L. Wang L. I. Xue S. J. Hu R. Jian R. J. Ho C. S. (2021). Gender differences of the improvement in balance control based on the real-time visual feedback system with smart wearable devices. Acta Bioeng. biomechanics 23 (1), 163–171. 10.37190/abb-01764-2020-02 34846031

[B57] Wolfson L. Whipple R. Derby C. A. Amerman P. Nashner L. (1994). Gender differences in the balance of healthy elderly as demonstrated by dynamic posturography. J. gerontology 49 (4), M160–M167. 10.1093/geronj/49.4.m160 8014390

[B58] Wood S. J. Paloski W. H. Clark J. B. (2015). Assessing sensorimotor function following ISS with computerized dynamic posturography. Aerosp. Med. Hum. Perform. 86 (12), A45–A53. 10.3357/AMHP.EC07.2015 26630195

[B59] Yagi C. Morita Y. Kitazawa M. Nonomura Y. Yamagishi T. Ohshima S. (2019). A validated questionnaire to assess the severity of persistent postural-perceptual dizziness (PPPD): the Niigata PPPD questionnaire (NPQ). Otology Neurotol. 40 (7), e747–e752. official publication of the American Otological Society, American Neurotology Society [and] European Academy of Otology and Neurotology. 10.1097/MAO.0000000000002325 PMC6641087 31219964

